# Changes in Alcohol-Based Handrub Usage Among Hospital Staff Four Years After the COVID-19 Pandemic: A Single-Centre Observational Time-Series Study

**DOI:** 10.3390/healthcare14020177

**Published:** 2026-01-09

**Authors:** Filip Waligóra, Anastazja Tobolewska-Kielar, Maciej Kielar

**Affiliations:** 1 Scientific Club of Medicover Hospital, General Surgery Clinic of Lazarski University, 02-972 Warsaw, Poland; anastazja10@yahoo.com; 2 Medicover Hospital, General Surgery Clinic of Lazarski University, 02-972 Warsaw, Poland; maciej.kielar@medicover.pl

**Keywords:** hand hygiene, disinfection, COVID-19, compliance, hospital-acquired infections

## Abstract

**Background/Objectives**: Alcohol-based handrub (ABHR) consumption is commonly used as an indirect proxy for hand hygiene practices. Hand hygiene compliance increased significantly during COVID-19, but sustainability remains uncertain. This study assessed ABHR consumption trends from 2022 to 2024 and compared them with pre-pandemic and pandemic-era rates. **Methods**: We conducted a follow-up observational study tracking quarterly ABHR consumption in a surgical department and hospital-wide (2022–2024). Consumption was normalized as mL per patient-day and compared with 2019–2020 data. Time-series regression with Newey–West standard errors assessed temporal trends. **Results**: Surgical department consumption declined 27.5% (55.9 to 40.5 mL/patient-day), returning to 2019 pre-pandemic levels. Hospital-wide consumption increased 36% (36.4 to 49.6 mL/patient-day). Neither trend reached statistical significance (*p* > 0.05). The 2024 surgical rate remained substantially below the 2020 pandemic peak (320 mL/patient-day). **Conclusions**: Pandemic-era ABHR consumption gains were not sustained in the surgical department despite maintained educational infrastructure, accessible dispensers, and consistent staffing. The critical missing element was systematic monitoring and feedback. Institutions relying solely on passive education may experience erosion of hand hygiene compliance post-crisis, highlighting the need for active surveillance programs to maintain behavioral gains.

## 1. Introduction

Hospital-acquired infections (HAIs) remain a major public health concern due to their significant contribution to patient morbidity and mortality. According to the World Health Organization, the prevalence of HAIs ranges from 3.5 to 12% in high-income countries to 5.7–19.1% in low-income and middle-income countries [1], with even higher rates among high-risk groups such as patients in intensive care units. Hospital-associated, drug-resistant infections are estimated to account for 136 million cases annually. The average hospitalization rates due to such infections are approximately 3% in low-income countries, 6% in middle-income countries, and 11% in high-income countries [2].

Healthcare workers’ hands are the most common vector for cross-transmission of pathogens between patients. Although both handwashing and sanitization are well-documented, effective preventive strategies, compliance with hand hygiene protocols remains suboptimal in many healthcare facilities, despite evidence that infection prevention and control measures can reduce HAIs by 35% to 70% [3]. These complications can increase length of hospital stay, morbidity and cost of treatment [4].

Hand hygiene by healthcare workers is widely recognized as the single most important measure to prevent HAIs [1,5]. The World Health Organization’s “Clean Care is Safer Care” campaign, launched in 2005, was designed to improve awareness and implementation of hand hygiene as a key strategy in reducing HAIs. The WHO multimodal hand hygiene improvement strategy consists of five core elements: system change (ensuring alcohol-based handrub availability at point of care), education and training, monitoring and performance feedback, workplace reminders, and institutional safety climate [1,5]. This comprehensive approach has demonstrated effectiveness across diverse healthcare settings globally [5,6,7].

Despite evidence supporting multimodal approaches, sustaining high levels of hand hygiene compliance remains challenging [1,8,9]. Research has shown that maintaining improvements often requires ongoing evaluation and performance feedback [10]. The average hand hygiene compliance rate has been reported as 40% in high-income countries and less than 20% in low-income countries [1].

During the COVID-19 pandemic, multiple centres reported marked increases in hand hygiene compliance; however, this effect was often transient and diminished over time, particularly when active monitoring and feedback were reduced [11,12,13]. Prior to the outbreak, adherence to hand hygiene protocols among healthcare workers had been suboptimal in many settings [14].

A 2021 audit-based study showed that hand hygiene compliance in Medicover Hospital in Warsaw initially stood at 49%. After the implementation of comprehensive educational interventions, compliance increased to 81% in a semi-annual audit, and slightly decreased to 77% during a final audit conducted one year later [14].

The objective of this study was to assess alcohol-based handrub (ABHR) consumption as an indirect proxy for hand hygiene compliance among hospital staff in Warsaw Medicover Hospital and to compare it with the rates observed before and during the COVID-19 pandemic. The comparison is based on data reported in the study *“The COVID-19 pandemic as a factor of hospital staff compliance with the rules of hand hygiene: assessment of the usefulness of the ‘Clean Care is a Safer Care’ program as a tool to enhance compliance with hand hygiene principles in hospitals”* [14]. The goal was to determine whether the improved compliance achieved during the pandemic has been sustained or whether continuous educational efforts similar to those implemented during the pandemic are necessary to maintain high adherence to hand hygiene protocols.

## 2. Materials and Methods

### 2.1. Study Setting and Design

Medicover Hospital in Warsaw is a 175-bed multidisciplinary facility providing general surgery, urology, cardiac surgery, anesthesiology and intensive care, gynecology, neonatology, cardiology, internal medicine, and pediatrics. This follow-up observational study extended our previous 2019–2020 analysis to assess post-pandemic trends (2022–2024). No changes in bed capacity or facility operations occurred during the observation period.

### 2.2. Data Collection and Measurement

ABHR consumption data were obtained from hospital supply records for both the surgical department (general surgery ward only; operating theaters excluded) and hospital-wide. Department nurses submitted weekly ABHR requisitions, which were fulfilled and recorded by procurement staff. Weekly data were aggregated quarterly and normalized per patient-day to account for occupancy variations.

All departments used identical ABHR formulation (Desmanol Pure, 70–90% propan-2-ol) throughout the study. Data represent volumes dispensed for dispenser refills; no products were returned. Data accuracy was verified through cross-referencing requisitions with warehouse records. We acknowledge the absence of independent verification as a limitation of the retrospective design.

Consumption rates were expressed as mL per patient-day and L per 1000 patient-days, consistent with World Health Organization (WHO) recommendations [3].

### 2.3. Statistical Analysis

Time-series regression assessed temporal trends in ABHR consumption. The model included time (quarterly units), service type (surgical vs. hospital-wide), and time × service interaction. In the regression model, hospital-wide data served as the reference category; the intercept represents the baseline hospital-wide ABHR consumption rate at the start of the observation period, the time coefficient reflects the quarterly change in hospital-wide consumption, the department coefficient represents the baseline difference for the surgical department, and the time × department interaction captures the additional quarterly change in the surgical department relative to hospital-wide services. Newey–West HAC standard errors (lag = 2) addressed potential autocorrelation and heteroskedasticity.

Regression coefficients (β), 95% confidence intervals (CIs), standard errors (SE), and *p*-values were reported for all model terms. A two-sided *p*-value < 0.05 was considered statistically significant. Model fit was assessed using R^2^. Analyses were performed in R version 4.5.1 (R Foundation for Statistical Computing, Vienna, Austria).

### 2.4. Ethical Considerations

According to Polish law (Act of 5 December 1996 on the Professions of Physician and Dentist), ethical approval is required for medical experiments involving human subjects. This study analyzed only aggregated, de-identified administrative data collected as part of routine infection control monitoring, without any patient-level information. It was conducted as a quality improvement initiative and therefore did not require review by a bioethics committee.

## 3. Results

**Figure 1:**
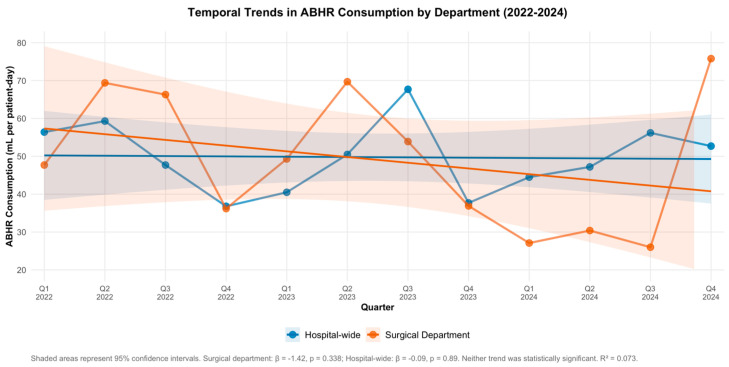
Temporal trends in alcohol-based handrub consumption by department, 2022–2024. Quarterly consumption rates (mL per patient-day) are shown for the surgical department (orange) and hospital-wide (blue). Points represent observed quarterly values; lines with dots connect consecutive observations; lines without dots represent fitted linear regression trend lines; shaded areas represent 95% confidence intervals for linear trend lines. The surgical department demonstrated a declining trend (β = −1.42 per quarter for time × department interaction, SE = 1.45, *p* = 0.338), while hospital-wide consumption showed minimal change (β = −0.09 per quarter, SE = 0.62, *p* = 0.89). Neither trend was statistically significant. Model R^2^ = 0.073, indicating that time and department explained only 7% of variance in ABHR consumption patterns.

A total of 24 quarterly observations were analyzed, comprising 12 time points for the surgical department and 12 for the hospital-wide dataset (Table A1 and Table A2). The primary time-series regression model included time (in quarters), department (surgical vs. hospital-wide), and a time × department interaction term, using Newey–West robust standard errors (lag = 2) to account for potential autocorrelation (Figure 1).

The model revealed no statistically significant overall trend in ABHR consumption across the hospital over time (β = −0.09, SE = 0.62, *p* = 0.89; 95% CI: −1.3 to 1.13). The surgical department showed a numerically higher baseline level of consumption, though this difference was not statistically significant (β = 7.11, SE = 6.93, *p* = 0.318; 95% CI: −6.48 to 20.7). The time × department interaction term indicated a steeper downward trend in the surgical department, though this was also non-significant (β = −1.42, SE = 1.45, *p* = 0.338; 95% CI: −4.26 to 1.41). The model intercept was statistically significant (β = 50.24, SE = 4.8, *p* < 0.001; 95% CI: 40.84 to 59.65), representing the estimated baseline consumption rate for hospital-wide services at the start of the observation period. The overall model fit was low (R^2^ = 0.073), indicating that time and department type explained only 7% of the variance in ABHR consumption, suggesting that other unmeasured factors may have influenced consumption patterns (Table 1).

Annual summaries of the quarterly data are presented to illustrate overall trends (Table 2 and Table 3). In 2022, the surgical department recorded 3577 patient-days and 200 L of ABHR consumption, corresponding to 55.9 mL per patient-day. Hospital-wide consumption in 2022 totaled 1157 L across 31,712 patient-days, yielding 36.4 mL per patient-day.

In 2023, surgical consumption decreased to 52.6 mL per patient-day (170 L over 3226 patient-days), while hospital-wide consumption increased to 48.9 mL per patient-day (1663 L over 33,940 patient-days).

By 2024, surgical department consumption further declined to 40.5 mL per patient-day (180 L over 4437 patient-days), while hospital-wide consumption stabilized at 49.6 mL per patient-day (1539 L over 31,014 patient-days).

To contextualize the 2022–2024 trends, these values can be compared to consumption rates from our previous study covering 2019–2020 [14]. In the surgical department, pre-pandemic consumption in 2019 was 40 mL per patient-day, which increased dramatically to 320 mL per patient-day during the pandemic peak in 2020—an 8-fold increase. The 2024 rate of 40.5 mL per patient-day represents a return to pre-pandemic baseline levels. Hospital-wide consumption data for 2019–2020 were not separately available in the previous study. The intervening period (2021) was not systematically documented, representing a limitation of this longitudinal analysis. Nevertheless, the available data clearly demonstrate that pandemic-era improvements in ABHR consumption were not sustained in the surgical department, with consumption returning to 2019 baseline by 2024 (Table A3).

Overall, the descriptive statistics reveal a progressive decline in ABHR consumption within the surgical department across the study period, while hospital-wide usage showed a moderate increase followed by stabilization. Although these temporal changes did not reach statistical significance, the observed trends suggest differing post-pandemic compliance patterns between departments.

## 4. Discussion

This study revealed divergent post-pandemic ABHR consumption patterns: progressive decline in the surgical department (returning to pre-pandemic baseline) and moderate increase hospital-wide. Neither trend achieved statistical significance, and the surgical department rate remained far below pandemic-era peaks [14].

Several operational factors at Medicover Hospital remained stable during the 2022–2024 observation period. There were no changes in hospital management or directorship, staff-to-patient ratios were maintained at levels defined by internal requirements, and ABHR dispenser infrastructure was unchanged, with dispensers consistently located at every bedside throughout the study period. The same ABHR formulation (Desmanol Pure, 70–90% propan-2-ol) was used throughout, ensuring product consistency.

Hand hygiene education was maintained through multiple channels. All newly hired staff received comprehensive hygiene training during orientation. Reminder campaigns reinforcing WHO hand hygiene principles were distributed via email, and posters illustrating the Five Moments for Hand Hygiene were displayed at workstations throughout the hospital. Additionally, all staff were required to complete annual refresher training via an e-learning platform with a mandatory competency test.

However, systematic direct observation of hand hygiene compliance was not implemented during this period, and no formal feedback mechanisms were in place to provide individual staff or departments with performance data on their actual hand hygiene behavior. Consumption data were tracked administratively but were not analyzed or communicated to staff in real-time as a performance metric. Therefore, while educational infrastructure and passive reminders were present, active monitoring and performance feedback components identified in the literature as critical for sustained compliance were absent [10,15].

The observed decline in surgical department consumption, despite maintained educational activities, highlights an important distinction in the hand hygiene literature: passive education versus active monitoring with feedback. At Medicover Hospital during 2022–2024, passive educational elements were present (posters, annual e-learning, email reminders), but active surveillance and feedback were absent. This represents a common approach in many healthcare institutions, where education is prioritized due to lower resource requirements compared to systematic monitoring.

However, the effectiveness of passive education alone appears limited. Novák et al. [16] reported that even with proper training, healthcare workers may fail to apply hand hygiene practices consistently in real-world scenarios, highlighting the “knowing-doing gap”, i.e., the disconnect between knowledge and behavior. Staff at Medicover Hospital received annual training and passed competency tests, demonstrating knowledge of hand hygiene principles. Yet consumption declined, suggesting that knowledge alone does not guarantee sustained behavioral compliance.

The literature increasingly suggests that education creates necessary knowledge but is insufficient for sustained behavior change without accountability mechanisms [10]. Monitoring and feedback provide this accountability by making hand hygiene performance visible and creating consequences (positive or negative) for behavior. In the absence of monitoring, hand hygiene becomes an “invisible” behavior with no external reinforcement, relying entirely on intrinsic motivation and professional conscientiousness—which the evidence suggests erodes over time without institutional support.

A study from Finland demonstrated sustained improvements in hand hygiene compliance over six years. The key factors were increased ABHR availability combined with regular direct observation and immediate feedback [15]. This provides an important contrast to the Medicover Hospital experience. Both institutions had optimal infrastructure (accessible ABHR dispensers) and educational programs. However, the Finnish institution implemented systematic observational monitoring with feedback, while Medicover Hospital did not.

The divergent outcomes—sustained compliance in Finland versus declining consumption at Medicover—suggest that the presence or absence of monitoring and feedback may be a critical differentiator. This is consistent with behavioral psychology principles: behaviors that are measured and reinforced tend to be sustained, while unmeasured behaviors tend to erode over time. The comparison suggests that passive educational reminders (posters, annual training) may be insufficient to maintain compliance without active monitoring that makes hand hygiene performance visible and actionable.

Effective monitoring and feedback are critical components of successful hand hygiene protocols. However, there is no universally accepted or standardized method for monitoring hand hygiene compliance in hospital settings. Several strategies exist, each with its own strengths and limitations.

The most commonly used method is direct observation, which is widely regarded as the gold standard. Nevertheless, this approach is susceptible to the Hawthorne effect, whereby individuals modify their behavior when they are aware of being observed, potentially leading to biased results [17]. Despite these limitations, observational monitoring combined with timely feedback has been shown to improve and sustain compliance over time [15].

An alternative approach involves the use of electronic monitoring systems (such as sensor-based technologies to record the frequency and volume of ABHR dispensed, or real-time locating systems to detect healthcare worker location and dispenser activation), which can provide automated and continuous data collection. These systems are generally more objective and less prone to observational bias, offering advantages such as larger datasets, real-time feedback, and the potential to enhance compliance. However, cost remains a major barrier to widespread implementation [18,19,20].

While electronic systems are promising, they have some limitations. Unlike direct observation, they may lack detailed contextual information about the behavior of healthcare staff, such as technique or timing. Therefore, current electronic systems may be best viewed as a complementary tool rather than a replacement for traditional methods.

According to WHO guidelines, hand hygiene—through either handwashing or the use of disinfectants—should be performed in accordance with the “Five Moments for Hand Hygiene” [5,21]. At Medicover Hospital, neither direct observational monitoring nor electronic systems were implemented during the 2022–2024 study period. While consumption data were tracked administratively, these data were not analyzed or communicated to staff as performance feedback. Therefore, staff had no objective information about their hand hygiene performance, and opportunities to identify and address declining compliance in specific departments or clinical areas were not available.

Multiple studies have documented that the sharp increase in hand hygiene compliance observed during the initial phase of the COVID-19 pandemic was not sustained long-term [12,13,14,22,23,24,25,26]. Our four-year observation at Medicover Hospital is consistent with this pattern. Compared to baseline levels from 2019 (40 mL per patient-day) and the peak during the pandemic in 2020 (320 mL per patient-day) [14], the 2022–2024 period shows a substantial post-pandemic decrease, with surgical department consumption returning to approximately pre-pandemic levels (40.5 mL per patient-day by 2024).

The initial pandemic-era increase likely resulted from multiple factors: heightened personal risk perception, intensive institutional emphasis, ubiquitous reminders, and widespread public health messaging. The subsequent decline occurred despite maintained educational infrastructure, suggesting that the heightened salience and personal threat perception associated with COVID-19 created a temporary crisis motivation [27]. Without systematic monitoring to maintain visibility and accountability, hand hygiene reverted to baseline patterns despite ongoing educational reminders.

These findings are particularly concerning given the broader context of antimicrobial resistance. Multidrug-resistant organisms (MDRO) are estimated to cause approximately 1.27 million deaths globally [28]. Pathogen transmission can occur not only through direct patient contact but also via medical equipment and contaminated surfaces. Hand hygiene remains a relatively simple yet essential measure in the prevention of HAIs [4,29].

At Medicover Hospital, several infrastructure-related factors can be ruled out: ABHR dispensers were universally accessible at bedside, staff-to-patient ratios were maintained at appropriate levels, there were no product shortages, and educational opportunities (orientation, annual training, reminders) were consistently available. This suggests that resource availability and basic knowledge were not limiting factors.

Instead, the observed decline may be attributable to factors related to behavioral sustainability and accountability. Without monitoring, hand hygiene behavior is invisible to leadership, peers, and individuals themselves, eliminating social accountability and removing opportunities for recognition of good performance or correction of poor performance. Staff received no data on their hand hygiene performance, eliminating opportunities for self-correction and improvement. Annual training assessed knowledge but not actual behavior. In the absence of measurement, hand hygiene may be deprioritized relative to other visible, measured aspects of clinical care. The heightened salience of hand hygiene during the COVID-19 pandemic dissipated over time, and without monitoring to maintain institutional emphasis, compliance drifted toward baseline levels. Staff likely retained knowledge of hand hygiene principles (as evidenced by passing annual competency tests) but translated this knowledge into behavior inconsistently in the absence of external accountability.

Beyond monitoring gaps, additional factors may have contributed to the observed decline. The literature reports time pressure, workflow demands, and professional role differences (with nurses typically demonstrating higher compliance than physicians [6,30,31,32]). However, without observational data or staff surveys, we cannot determine the relative importance of these factors at Medicover Hospital.

While these findings raise important questions, several methodological limitations must be acknowledged:

1. **Proxy measurement:** Consumption data cannot capture actual behavior, technique quality, timing appropriateness, or distinguish productive use from wastage.
2. **Statistical power:** Small sample size (*n* = 12 per department) and low model fit (R^2^ = 0.073) indicate unmeasured factors explain most variance.
3. **Educational engagement:** While we documented educational activities, we lacked data on staff engagement, knowledge retention beyond annual testing, or behavioral translation.
4. **Observational data:** Absence of direct observation precluded assessment of actual practices or identification of specific moments when hand hygiene was omitted.
5. **Clinical outcomes:** We did not measure HAI rates, MDRO prevalence, or surgical site infections, precluding assessment of patient safety impact.
6. **Qualitative data:** We lacked staff perspectives on barriers, attitudes, or responses to different approaches.
7. **Causality:** While monitoring absence coincided with consumption decline, causality cannot be definitively established; other unmeasured factors may have contributed.

The observed decline in ABHR consumption in the surgical department is concerning from an infection prevention perspective, as surgical patients are at elevated risk for healthcare-associated infections due to invasive procedures and disruption of natural barriers. However, this study did not examine clinical outcomes (infection rates, MDRO prevalence), and we cannot determine whether the consumption decline translated into increased infection risk. Future research should examine the relationship between consumption patterns and clinical outcomes to assess whether consumption thresholds exist below which patient safety is compromised.

## 5. Conclusions

ABHR consumption in the surgical department declined by 27.5% over three years, returning to pre-pandemic baseline levels despite maintained educational infrastructure and optimal resource availability. The critical missing component was systematic monitoring and feedback—mechanisms that create accountability and enable targeted intervention when compliance declines.

The contrast between our experience and sustained compliance achieved with active monitoring programs suggests that education creates necessary knowledge but monitoring and feedback sustain behavior. Institutions relying solely on periodic training and reminders may experience gradual compliance erosion, particularly after crisis-driven improvements.

Important limitations apply: consumption is an imperfect compliance proxy, and small sample size limits statistical power. Nevertheless, these findings suggest that evidence-based hand hygiene programs should integrate role-specific education, systematic compliance monitoring (observational or electronic), regular feedback to individuals and departments, integration into institutional quality metrics, and sustained leadership commitment with appropriate resources.

Future research should:

- Prospectively evaluate cost-effectiveness of different monitoring strategies;
- Compare observational versus electronic monitoring approaches;
- Examine optimal feedback formats and frequencies;
- Assess relationships between consumption patterns, observed compliance, and clinical outcomes;
- Investigate strategies for sustaining crisis-driven behavioral improvements;
- Explore healthcare worker perspectives on barriers and facilitators through qualitative research.

The post-pandemic period represents a critical moment for infection control globally. Active, ongoing monitoring and feedback appear necessary to maintain gains achieved during COVID-19 and prevent regression to pre-pandemic compliance levels.

## Figures and Tables

**Table 1:** Time-series regression analysis of ABHR consumption trends (2022–2024).

Parameter	β	SE	95% CI	*p*-Value
Intercept (hospital-wide baseline)	50.24	4.80	40.84 to 59.65	<0.001
Time (per quarter)	−0.09	0.62	−1.30 to 1.13	0.89
Department (surgical vs. hospital-wide)	7.11	6.93	−6.48 to 20.70	0.318
Time × Department interaction	−1.42	1.45	−4.26 to 1.41	0.338

Model R^2^ = 0.073; Newey–West HAC standard errors (lag = 2). Abbreviations: ABHR, alcohol-based hand rub; β, regression coefficient; SE, standard error; CI, confidence interval; HAC, heteroskedasticity and autocorrelation consistent.

**Table 2:** ABHR Consumption Rates—Hospital-wide (2022–2024).

Year	Patient-Days	mL/Patient-Day	L/1000 Patient-Days
2022	31,712	36.4	36
2023	33,940	48.9	49
2024	31,014	49.6	50

Abbreviations: mL, milliliters; L, liters; 1 L/1000 patient-days = 1 mL/patient-day.

**Table 3:** ABHR Consumption Rates—Surgical Department (2022–2024).

Year	Patient-Days	mL/Patient-Day	L/1000 Patient-Days
2022	3577	55.9	56
2023	3226	52.6	53
2024	4437	40.5	41

Abbreviations: mL, milliliters; L, liters; 1 L/1000 patient-days = 1 mL/patient-day.

## Data Availability

The quarterly, aggregated ABHR consumption data supporting the findings of this study are provided in Table A1 and Table A2 in the Appendix A.

## Abbreviations

The following abbreviations are used in this manuscript:

ABHR	Alcohol-based handrub
HAIs	Hospital-acquired infections
WHO	World Health Organization
MDRO	multidrug-resistant organisms

## Appendix A

**Table A1:** Quarterly ABHR consumption rates in the surgical department, 2022–2024.

Year	Quarter	Patient-Days	Disinfectant (L)	mL/Patient-Day
2022	Q1	839	40	47.7
	Q2	1080	75	69.4
	Q3	830	55	66.3
	Q4	828	30	36.2
2023	Q1	811	40	49.3
	Q2	861	60	69.7
	Q3	742	40	53.9
	Q4	812	30	36.9
2024	Q1	1109	30	27.1
	Q2	986	30	30.4
	Q3	1155	30	26
	Q4	1187	90	75.8

Abbreviations: mL, milliliter; L, liters.

**Table A2:** Quarterly ABHR consumption rates hospital-wide, 2022–2024.

Year	Quarter	Patient-Days	Disinfectant (L)	mL/Patient-Day
2022	Q1	7499	423	56.4
	Q2	8479	503	59.3
	Q3	7963	380	47.7
	Q4	7771	286	36.8
2023	Q1	8636	350	40.5
	Q2	8517	430	50.5
	Q3	8347	565	67.7
	Q4	8440	318	37.7
2024	Q1	8654	385	44.5
	Q2	8268	390	47.2
	Q3	6054	340	56.2
	Q4	8038	424	52.7

Abbreviations: mL, milliliter; L, liters.

**Table A3:** ABHR consumption rates in the surgical department: baseline year 2019 vs. pandemic peak (2020) and post-pandemic period (2022–2024).

Year	mL/Patient-Day	L/1000 Patient-Days	Change vs. 2019 (%)
2019	40.0	40	0.0
2020	320.0	320	+700.0
2022	55.9	56	+39.8
2023	52.6	53	+31.5
2024	40.5	41	+1.3

The 2019–2020 values were obtained from the previously published study [14]. Abbreviations: mL, milliliters; L, liters; 1 L/1000 patient-days = 1 mL/patient-day.
